# Global Migration Dynamics Underlie Evolution and Persistence of Human Influenza A (H3N2)

**DOI:** 10.1371/journal.ppat.1000918

**Published:** 2010-05-27

**Authors:** Trevor Bedford, Sarah Cobey, Peter Beerli, Mercedes Pascual

**Affiliations:** 1 Department of Ecology and Evolutionary Biology, University of Michigan, Ann Arbor, Michigan, United States of America; 2 Howard Hughes Medical Institute, University of Michigan, Ann Arbor, Michigan, United States of America; 3 Department of Scientific Computing, Florida State University, Tallahassee, Florida, United States of America; Imperial College London, United Kingdom

## Abstract

The global migration patterns of influenza viruses have profound implications for the evolutionary and epidemiological dynamics of the disease. We developed a novel approach to reconstruct the genetic history of human influenza A (H3N2) collected worldwide over 1998 to 2009 and used it to infer the global network of influenza transmission. Consistent with previous models, we find that China and Southeast Asia lie at the center of this global network. However, we also find that strains of influenza circulate outside of Asia for multiple seasons, persisting through dynamic migration between northern and southern regions. The USA acts as the primary hub of temperate transmission and, together with China and Southeast Asia, forms the trunk of influenza's evolutionary tree. These findings suggest that antiviral use outside of China and Southeast Asia may lead to the evolution of long-term local and potentially global antiviral resistance. Our results might also aid the design of surveillance efforts and of vaccines better tailored to different geographic regions.

## Author Summary

Infections by the influenza A virus show highly seasonal patterns in temperate regions. Winter is flu season. Over the course of the autumn and winter, a small number of initial infections grow to encompass a significant proportion of the population. At the end of the winter, infection disappears. It has been suggested that the strains founding each temperate flu season originate from China and Southeast Asia, where influenza A is less seasonal. We test this hypothesis through analysis of genetic sequences from viruses sampled throughout the world between 1998 and 2009. We find that although China and Southeast Asia play the largest role in the migration network, temperate regions, particularly the USA, also make important contributions. Not all temperate strains of influenza die out with the end of the winter season. Instead many strains emigrate to more favorable climes. Thus, we find patterns of influenza transmission to be highly dynamic. Because of emigration out of temperate regions, mutations harbored by temperate strains of influenza A can spread to the global virus population. This means that regional public health decisions regarding influenza may have global impacts.

## Introduction

Yearly epidemics of influenza viruses are responsible for between 250,000 and 500,000 deaths globally, with influenza A causing the bulk of mortality and morbidity [1]. Influenza is concentrated in the autumn and winter in temperate regions, but shows less periodic transmission in the tropics. Influenza is not endemic to any particular region in the world but appears to be dynamically sustained; a local epidemic will sweep through a particular region, fade out and then be reseeded by contact with a different region's local epidemic the following year [2], [3]. Understanding the geographic structure of influenza transmission is of critical importance to our efforts to combat the disease. Here, we identify previously unreported genetic structure in the global population of seasonal influenza A (H3N2) viruses and show how this structure arises from the dynamics of the global transmission network. Whereas previous hypotheses propose a source-sink model of viral evolution, in which a network of populations in East and Southeast (E-SE) Asia seeds annual epidemics in temperate latitudes [3], [4], we find that strains of influenza often circulate outside Asia, sustained by complex migration dynamics. This persistence may have long-term effects on influenza's evolution. Through migration between regions, influenza may persist over time, even if no particular region serves as a reservoir of disease.

## Results

### Genetic diversity

Genetic diversity in influenza A (H3N2) is highly restricted and few unique hemagglutinin (HA) variants exist at any one time [5]. We find that 4355 sequences sampled from 1998 to 2009 show average nucleotide diversity 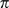 of 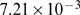 substitutions per site (95% confidence interval 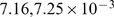) between pairs of contemporaneous sequences, defined as sequences sampled no more than 30 days apart from one another. This level of nucleotide diversity is approximately 15 times greater than that of human genes (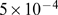) [6]. However, it is significantly lower than nucleotide diversity of HIV sequences isolated from a single patient (0.08) [7]. Despite showing limited genetic diversity, the virus evolves extremely quickly at a rate of 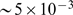 substitutions per site per year, resulting in rapid genetic turnover from year-to-year [4], [8].

At a continental scale, we find substantial geographic population structure in the influenza virus. We classified influenza samples into 7 regions: China (encompassing mainland China, Hong Kong, Macau and Taiwan), Europe, Japan, Oceania, South America, Southeast Asia (encompassing Cambodia, Indonesia, Malaysia, Myanmar, the Philippines, Singapore, Thailand, and Vietnam) and the USA. These regions were chosen based upon geography as well as sampling density (Table S1, Figure S1, Figure S2). On average, genetic diversity among contemporaneous sequences is greater between regions, 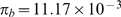 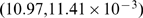, than within regions, 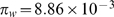 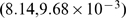 (Table S2). This distinction is commonly quantified as 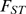, equal to 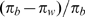 [9]. 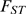 greater than 0 indicates genetic isolation among regions, referred to as population structure. In influenza, 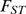 is 0.207 (0.134, 0.270). For comparison, continental genetic differences in humans show 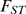 of 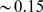 [10].

### Migration rates

To explain the genetic relationships among viral samples, we used a population genetic model based on the structured coalescent [11], [12], which describes the genealogical patterns connecting members of a reproducing population. This model explicitly incorporates sampling date [13] and sampling region [14] to reconstruct the genetic history of samples taken from an evolving population. In this analysis, we account for differences in the overall sampling resolution (Figure S1) and for differences in temporal sampling patterns (Figure S2) by taking 100 random subsamples from the available influenza A (H3N2) sequences. In each resampled replicate, the number of sequences from each region was the same. Our migration rate estimates represent the mean across these replicates. Confidence intervals were produced by comparing estimates across the replicate pool.

**Figure 1:**
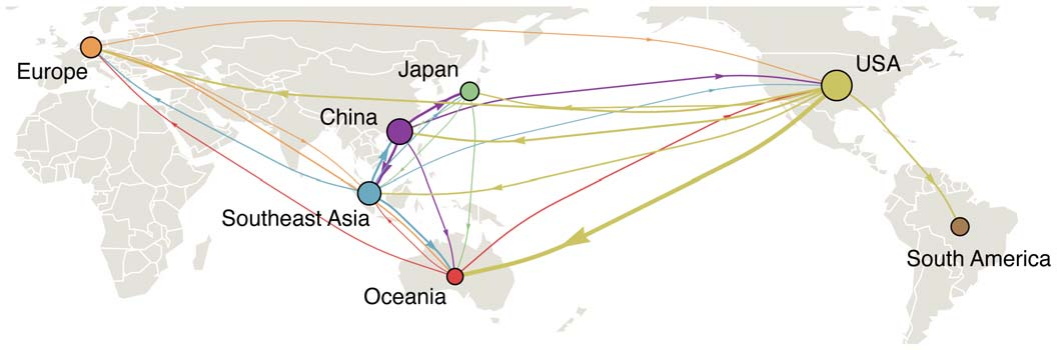
Global migration patterns of influenza A (H3N2) estimated from sequence data between 2002–2008. Arrows represent movement of influenza from one region to another, with arrow width proportional to the rate of migration of a single lineage of influenza. Arrows representing migration rates of less than 0.1 migration events per lineage per year were removed from the figure for clarity. Circle areas are proportional to a region's eigenvector centrality, a measure of the importance of a node in the migration network. The eigenvector centrality is equal to the expected stationary distribution when tracing the history of a lineage backwards in time [30].

The statistic 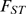 showed evidence of structure among influenza virus populations, arising from structure in the contact network of host populations. Our structured coalescent analysis goes further, revealing not only population structure, but also rates of migration of influenza viruses between regions (Figure 1). Migration rate estimates varied little over resampled replicates (Table S3) suggesting that sampling particulars had little influence over our coalescent results. In support of a global metapopulation model, we find that all regions act to some extent as sources in the migration network. We observe frequent gene flow from China into the USA, but also from the USA into China. Both in terms of overall rate of emigration and in terms of network centrality, China, Southeast Asia and the USA make the strongest contributions to the migration network (Table 1, Figure 1).

**Table 1:** Means and 95% confidence intervals across resampled replicates for the total rates of immigration from all other regions and emigration to all other regions for each region measured in terms of migration events per lineage per year.

	Immigration	Emigration
China	0.79 (0.49, 1.18)	1.05 (0.59, 1.73)
Europe	0.70 (0.52, 0.90)	0.59 (0.33, 1.06)
Japan	0.76 (0.57, 0.98)	0.51 (0.29, 1.14)
Oceania	1.27 (0.91, 1.81)	0.55 (0.31, 0.91)
South America	0.47 (0.42, 0.52)	0.30 (0.21, 0.38)
Southeast Asia	0.85 (0.52, 1.15)	0.91 (0.49, 1.64)
USA	0.70 (0.44, 1.11)	1.62 (0.91, 2.33)

The estimated migration network correlates well with the frequency of air travel between regions (compare Figure 1 to Figure 1 of Hufnagel et al. [15]). For example, South America is relatively isolated in the global aviation network [15], although it possesses more ample connections to North America. Consistent with this detail, we find that immigration of influenza into South America is rare and that when it occurs it most often comes from the USA. Additionally, influenza in China migrates most frequently to Japan and Southeast Asia, while influenza in Southeast Asia migrates most frequently to China and Oceania. Still, although the world has become tightly connected through travel, it appears that influenza sweeps through local populations fast enough to sustain substantial geographical population structure on a continental scale.

### Genealogical history

**Figure 2:**
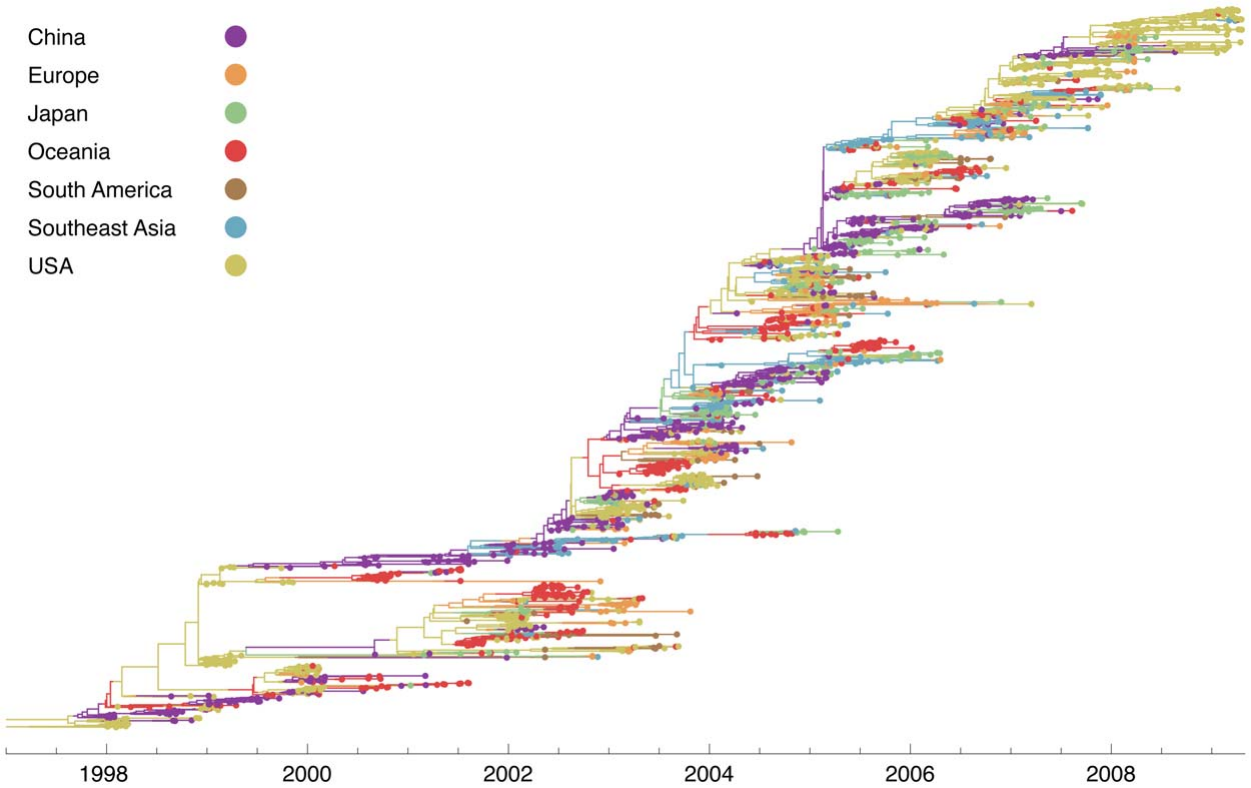
Genealogy of 2165 influenza A (H3N2) viruses sampled from 1998 to 2009. Each point represents a sampled virus sequence, and the color of the point shows the location where it was sampled. Samples are explicitly dated on the 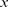-axis. Tracing a vertical line gives a contemporaneous cross-section of virus isolates. The genealogy is sorted so that lineages that leave more descendants are placed higher on the 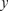-axis than other, less successful lineages. This sorting places the trunk along a rough diagonal, and it places lineages that are more genetically similar to the trunk higher on the 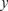-axis than lineages that are farther away from the trunk. The tree shown is the highest posterior tree generated by the Markov chain Monte Carlo (MCMC) procedure implemented in the software program Migrate v3.0.8 [14], [20].

A detailed genealogical history of the influenza A (H3N2) virus population was reconstructed through further analysis of a large number of sampled sequences (Figure 2). Here, temporal patterns are of predominant interest. Samples that are spread out over time provide the most information toward this goal. To reduce the computational complexity of the data set while retaining the most temporal information, we pruned the 4355 sequences to 2165 sequences by taking at most 10 sequences per month from each region. Sequence counts in richly sampled regions were reduced by this procedure, while regions with poor sampling were left mostly intact (Figure S2).

The influenza tree has the characteristic shape of a long trunk and short side branches [16], [17], resulting from the combined effects of temporal sampling and rapid coalescence. Looking backwards in time, two sorts of events occur: new lineages are sampled and existing lineages coalesce. If the rate of coalescence is large compared to the rate at which new lineages are sampled, then the genealogy will appear spindly with few contemporaneous lineages. The observed pattern of rapid coalescence arises from immune-driven adaptive evolution in the HA gene [5], [18], [19].

Contacts between regions produce migration events, which we depict as shifts in color in the virus genealogy (Figure 2). The genealogy shows that migration events between major geographic regions are uncommon, and thus the virus is not well-mixed among regions. Generally, we observe genetic diversification over the course of a regional epidemic, after which time few, if any, lineages persist. Local persistence appears in a genealogy as a side branch present in the same region over multiple seasons. It is clear from the influenza tree that this pattern is rare, suggesting lineages of influenza do not often persist from season to season within temperate regions.

While a general lack of local persistence is consistent with previous results [2], [3], we find that contrary to previous hypotheses [3], [4] seasonal epidemics in temperate regions can seed future epidemics around the world. For example, we find that the 1998–1999 USA epidemic seeds two major influenza lineages. The first of these lineages appears as a temperate lineage that circulates predominantly in Europe, Oceania, South America and the USA. This lineage persists for 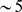 years. The second lineage is part of the trunk of the genealogy; it migrates from the USA into China, where it persists from 2000 to 2003. After 2003, this lineage spreads to the rest of the world. Thus, we find that global persistence is aided by metapopulation structure in which infection is dynamically sustained through contact between regions of different seasonality.

### Trunk reconstruction

At any given moment there is a strain of influenza that will eventually, through natural selection and genetic drift, become the progenitor of all future influenza strains. Looking backward in time, this is equivalent to the statement that all current strains of influenza share a common ancestor at some time in the past. This progenitor strain corresponds to the trunk of the influenza genealogy (Figure 2) and is where historically relevant evolution occurs; only mutations that occur along the trunk are maintained indefinitely, while mutations that occur along other branches will eventually be lost. Still, mutations to side branches may have important, if transitory, effects. Antigenically novel variants will be more likely than other strains to become the progenitors of the influenza population. However, minor variants arising in poorly connected regions of the world, i.e. South America, will be less likely to spread than variants arising in highly connected regions, i.e. the USA. Thus, even in the presence of antigenic drift, we expect migrational structure to play a role in which strain eventually takes over the influenza population.

**Figure 3:**
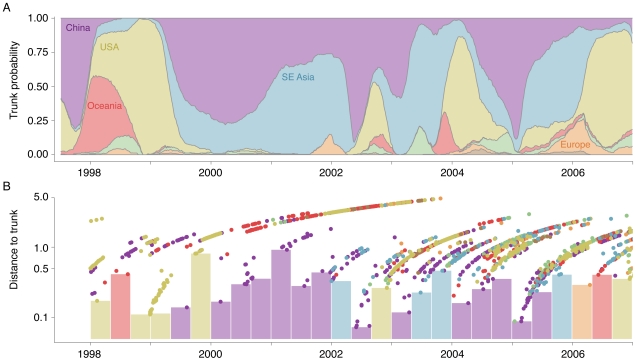
Estimation of the geographic location of the trunk of the influenza tree over time. (A) Probability that the trunk of the influenza tree exists in a particular region at a particular point in time. Trunks were obtained from sampled genealogies generated from the spatially and temporally tagged sequences. At each point in time, some sampled genealogies will have one region as the trunk, while other sampled genealogies will have a different region as the trunk. This plot encapsulates this uncertainty. At each point in time, the width of region represents the mean proportion from 0 to 100% of sampled genealogies bearing this region as trunk. At times when one color dominates the 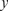-axis, we can be fairly certain that the trunk of the genealogy is in this location. Other times, when there is a mix of colors, we are not so certain. (B) Distance to the trunk, measured in terms of years, for each sampled influenza sequence. Here, points represent individual tips of the influenza tree colored as in Figure 2. The height of each point on the 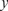-axis shows the mean distance to the trunk across the full range of estimated genealogies. Bars identify the closest sample to the trunk within a 4 month window of time. Bars are colored according to regions of these samples.

Our structured coalescent approach explicitly models the location of the genealogy trunk over time, allowing direct calculation of the proportion of the trunk belonging to each geographic region (Table 2, Figure 3). Consistent with previous predictions [3], we find that from 1998 to 2007 the trunk of the genealogy predominantly resides in China (34%) and Southeast Asia (32%). However, a significant proportion of the trunk resides in the USA (24%). As previously established, migration patterns near the tips of the tree support China, Southeast Asia and the USA as source populations (Figure 1). We would expect that as source populations, these regions would predominate the trunk of the genealogy. These results demonstrate that the last 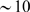
years of historically meaningful evolution in the virus population occurred primarily in China, Southeast Asia and the USA.

**Table 2:** Means and 95% credible intervals over sampled genealogies for the location of the genealogy trunk between the years of 1998 and 2007.

	Trunk proportion
China	0.34 (0.10, 0.58)
Europe	0.02 (0.00, 0.09)
Japan	0.03 (0.00, 0.11)
Oceania	0.05 (0.00, 0.13)
South America	0.00 (0.00, 0.06)
Southeast Asia	0.32 (0.08, 0.53)
USA	0.24 (0.12, 0.39)

## Discussion

We have shown that the genetic population structure of influenza A (H3N2) arises in part from global migration dynamics, with the most important contributions from China and Southeast Asia, but nonetheless significant contributions from temperate regions outside Asia (Figure 1). In contrast to the prevailing source-sink model, we find evidence of significant migration of viruses from temperate regions to tropic regions, and that lineages may exist outside of Asia for several seasons, persisting through dynamic migration between regions of different seasonality (Figure 2). Additionally, we find that China, Southeast Asia and the USA all contribute to the trunk of the influenza genealogy (Figure 3), and thus mutations occurring within these regions have shaped the global flu population. The evolution of H3N2 influenza over the past 10 years thus reflects the dynamics of a global metapopulation, rather than a metapopulation restricted to East and Southeast Asia.

Our use of the structured coalescent model to analyze influenza evolution represents a significant step forward over previous techniques. The tree constructed by Russell et al. [3] using phylogenetic methods, is a single estimate of the HA genealogy. We use a Bayesian sampling technique to analyze a large number of trees concordant with the genetic data [20]. More importantly, our coalescent method explicitly incorporates sampling date, sampling location and also an underlying model of the demographic process. These details provide substantially more context, and thus allow for more accurate reconstruction. Rambaut et al. [4] use a similar Bayesian coalescent approach; however, their technique did not take into account population structure.

By analyzing a large number of sampled trees and through resampled replicates, we establish the degree of uncertainty of our estimates. Each migration rate has a confidence interval attached to it (Table S3). Additionally, our estimates of the trunk location over time have a degree of confidence associated with them. Our statistical model strongly suggests that the 1998–1999 USA epidemic forms the trunk of the influenza genealogy (Figure 3A). Consistent with this hypothesis, we observe that samples from the USA during this period coalesce to the trunk of the genealogy rapidly in absolute terms, not just relative to other samples (Figure 3B). From 2000 to 2002, Chinese samples are closer to the trunk than samples from the USA and Oceania, but are not close to the trunk in absolute terms (Figure 3B). Because of this, there is considerable uncertainty as to whether the trunk of the genealogy resides in China or in Southeast Asia (Figure 3A). This particular result is especially supportive of our method, as we lack samples in Southeast Asia prior to 2002 (Figure S2), yet still we infer that Southeast Asia may be contributing to the trunk of the genealogy. There are other time periods (e.g. 2006) in which samples are far from the trunk, suggesting the possibility that the trunk may be located outside of sampled regions.

Regardless of methodological differences and differences of interpretation, our results are compatible with the results of Russell et al. [3] and Rambaut et al. [4]. In their analysis of mean distance to the trunk, Russell et al. find that the USA is behind China, Taiwan, Hong Kong and South Korea, but ahead of every other country sampled, including all of the Southeast Asian countries. This itself should suggest that the USA plays an important role in the global migration dynamics. Additionally, the inference of the 1998–1999 USA epidemic as the trunk of the genealogy is congruent with the findings of Rambaut et al. In a genealogy produced from only USA sequences (their Supplementary Figure 3e), it is clearly seen that while most USA epidemics occur as side branches, the 1998–1999 epidemic is distinctly part of the trunk of the genealogy. Russell et al. state: “the tree does show evidence for bidirectional seeding but no evidence for non-E&SE Asian strains contributing to long-term evolution of the virus during the study period.” We suggest that if Russell et al. had analyzed samples from 1998–1999, they would have obtained different results.

It is possible, for example that some of the strength of the USA's contribution to the migration dynamics (Figure 1) comes from its proximity to the Central American tropics. In this scenario, gene flow back and forth across the Pacific would be attributable to strains of influenza circulating in Central America that pass through the USA in their spread to the rest of the world. However, if this scenario were true, we might expect that the Central American influence would extend to South America in addition to the USA. We do not see evidence of this; South America contributes the least among studied regions to the global migration dynamics.

**Figure 4:**
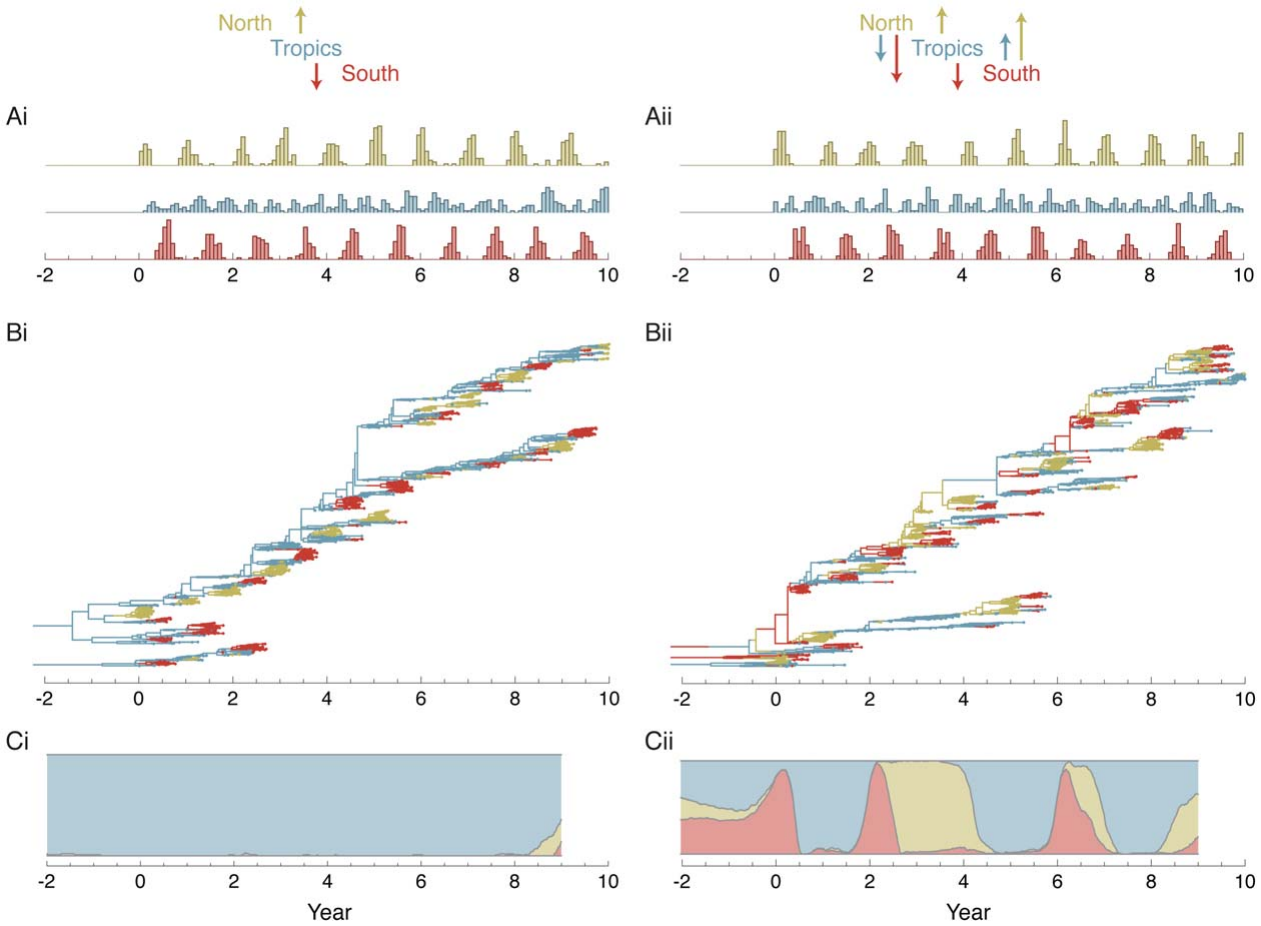
Analysis of epidemiological simulations for a source-sink model (i) and an equal contacts model (ii) of spatial structure. (A) Histogram of sampled sequence dates. Five hundred sequences were sampled randomly from each deme of the simulated virus population over a 10 year time period in proportion to abundance. The seasonality of the North and the South are reflected in the temporal sampling patterns. (B) Inferred genealogy from sampled sequences. Each point represents a sampled virus sequence, and the color of the point shows the location where it was sampled. The genealogy shown represents the highest posterior tree. (C) Inferred location of the trunk of the genealogy over time. Trunks were obtained from a posterior sample of genealogies by taking a random lineage present between years 9 and 10 and tracing its ancestry backward in time. Uncertainty of the trunk location is captured by this methodology. In times when one color dominates the 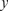-axis, we can be fairly certain that the trunk of the genealogy is in this location. Other times, when there is a mix of colors, we are not so certain.

Additional evidence for a temperate contribution to the migration network and to the trunk of the genealogy comes from epidemiological simulations (see Materials and Methods). In simulations with equal rates of contact between hosts in a northern population, a tropical population and a southern population we observe that despite strong seasonality in the temperate regions, substantial emigration occurs out of the temperate populations (Table 3). In this scenario, we find that although the trunk of the genealogy resides predominantly in the tropics, it often passes through the temperate populations during the course of a seasonal epidemic (Figure 4).

**Table 3:** Mean estimates and 95% credible intervals for effective population size 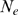, rate of migration 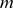 and trunk proportion for each region in simulated data sets based on 500 samples from each deme over a 10 year period.

		Source-sink model	Equal contact model
Population size	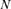	113.6 (93.0, 140.0)	122.0 (95.2, 162.4)
	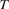	219.3 (192.2, 250.2)	220.7 (183.6, 283.8)
	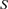	166.3 (141.2, 193.8)	163.9 (136.0, 208.8)
Migration rate	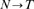	0.06 (0.02, 0.13)	0.38 (0.23, 0.50)
	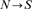	0.01 (0.00, 0.06)	0.38 (0.26, 0.49)
	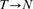	0.37 (0.30, 0.47)	0.38 (0.29, 0.52)
	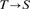	0.38 (0.31, 0.45)	0.37 (0.29, 0.48)
	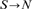	0.02 (0.00, 0.06)	0.39 (0.21, 0.53)
	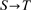	0.02 (0.00, 0.09)	0.37 (0.18, 0.50)
Trunk proportion	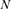	0.01 (0.00, 0.06)	0.24 (0.13, 0.39)
	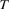	0.99 (0.94, 1.00)	0.55 (0.39, 0.69)
	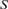	0.00 (0.00, 0.01)	0.21 (0.07, 0.39)

Examining the influenza genealogy (Figure 2), it is apparent that regional outbreaks often result from very few immigration events, consistent with previous results [2]. For example, the 2003 epidemic in Oceania appears almost completely monophyletic and can trace its history to a single migration event (or perhaps multiple migration events of identical strains) in early 2003. Thus, even if there are millions of infected individuals at the peak an epidemic, the genetic diversity of the virus will be bottlenecked at the beginning of the outbreak [21]. The bottlenecking effect of low migration may have contributed to the observed pattern of restricted genetic diversity in influenza. We observe similar effective population sizes across all regions (Table S4), consistent with the hypothesis that epidemic influenza is passed from one region to another, persisting nowhere. If there were a reservoir of endemic influenza in E-SE Asia (or elsewhere) that repeatedly seeded epidemics in the rest of the world, then the coalescence of E-SE Asian lineages would not be bottlenecked to the same extent, in which case we would observe significantly deeper coalescence events in this region and a correspondingly larger effective population size. This is not, however, the pattern we observe, reinforcing the idea that a metapopulation structure underlies influenza's persistence, even in the tropics [3], [4].

The global dynamics of influenza virus population influence a variety of public health decisions. Because influenza frequently migrates out of the USA, seeding epidemics in other parts of world, actions taken to combat the disease in the USA can have global impacts. For instance, the use of antivirals in the USA may promote the evolution of drug-resistant strains, which could then spread to the rest of the world. And conversely, vaccination programs outside of E-SE Asia have the potential to curb the global spread of the disease. Additionally, with increased knowledge of the patterns of flu migration, it may be possible to tailor vaccine design to particular areas of the globe. For instance, we observe that most influenza in South America arrives from the USA. This suggests that vaccines used in South America should be preferentially constructed from the USA strains of the previous season.

Our research suggests that the majority of historically relevant evolution of the influenza virus occurs in China, Southeast Asia and the USA, with other regions of the globe playing significant, but relatively minor roles. This conclusion is, to some extent, contingent upon the restricted temporal and spatial patterns of viral sampling. There may be other regions of the world, such as Africa, Central America and India, that act as important sources in the worldwide influenza migration network. Increased worldwide sampling of the influenza virus would further clarify the complex migration dynamics of the virus.

## Materials and Methods

### Sequence data and diversity

Sequences belonging to the HA1 domain of the hemagglutinin (HA) gene were downloaded from the Influenza Virus Resource of GenBank [22]. Only non-lab strains of at least 900 bases with fully specified dates (day, month, year) and countries of origin were used. We restricted our analysis to sequences dated from 1998 to 2009. We categorized the resulting 4355 samples into 7 geographic regions (Figure S1, Figure S2). Regions were chosen with the intention of maximizing geographic distinctions while simultaneously maintaining enough samples to make accurate regional inferences. Sequences were aligned using MUSCLE v3.7 under default parameters [23]. Nucleotide diversity, measured in terms of substitutions per site, was calculated as the mean proportion of mismatches across all contemporaneous pairs of sequences. Only sequences whose sample dates were within 30 days of each other were considered contemporaneous. To avoid bias toward well sampled regions, the overall within-region nucleotide diversity was estimated as the average of region-specific diversity estimates:
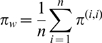
where 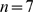 regions and 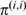 refers to diversity estimates where both samples in each pair are from region 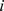 (Table S2). The overall between-region diversity was estimated in a similar fashion:
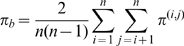
where 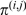 refers to diversity estimates where one sample is from region 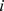 and the other sample is from region 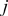. Confidence intervals were estimated by taking 1000 bootstrap replicates from the total pool of sequences. We caution that our estimates of diversity may be over-estimates due to the fact that strains are often first characterized by HI cross-reaction; antigenically novel strains are then preferentially sequenced. If the bias towards antigenically novel strains is similar in each region, then we would expect estimates of 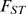 to be robust to this effect as 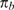 and 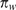 should be biased equally.

### Coalescent estimation of migration rates

Evolutionary dynamics were estimated using a Bayesian Markov chain Monte Carlo (MCMC) approach. MCMC explores the parameter-space through a random walk, converging on the posterior distribution of the model parameters. Evolutionary parameters shared across locations were estimated using the MCMC techniques implemented in the coalescent inference program BEAST v1.4.8 [24]. Here, trees are constructed following a single-population coalescent process, which imposes a prior on the branch lengths of the tree. We used the HKY85 model [25] to parameterize the mutational process, with equilibrium nucleotide frequencies taken from observed nucleotide frequencies, and the evolutionary rate across sites held constant. The transition/transversion ratio 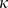 was estimated to be 6.745 (95% credible interval 6.267–7.269). The rate of nucleotide substitution 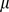 was estimated to be 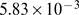 substitutions per site per year (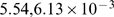 substitutions per site per year).

These mutational parameters were held constant in subsequent analyses to estimate coalescent parameters for each geographic region via a similar MCMC technique implemented by Migrate v3.0.8 [14], [20] that allows joint analysis of multiple regions. Henceforth, we refer to these sampling regions as demes. Migrate estimates the parameter 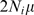, where 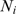 is the effective population size of deme 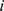. We measure 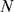 in terms of years, rather than generations, corresponding to our measurement of 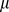 in terms of substitutions per site per year. Thus, 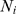 measures the expected number of years for two samples from within a deme to coalesce into a single lineage. We call this the ‘timescale of coalescence’ of deme 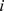. The prior distribution of 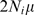 was assumed to be exponential with a mean of 0.1 substitutions per site. Migrate estimates the rate of migration 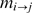 via the parameter 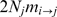. The rate of migration 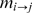 is measured in terms of migration events from deme 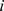 into deme 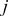 per lineage per year. The prior distribution of 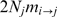 was assumed to be exponential with a mean of 0.1 migration events.

To confirm that sampling patterns did not influence our results, we performed independent analyses of 100 resampled replicates. For each replicate, we limited each region to the same number of samples between the years 2002 and 2008 during which time each region was well represented (Figure S2). South America had only 61 samples during this span of time, and so sample counts in other regions were constrained to match. Migration rate estimates varied little across the 100 resampled replicates, suggesting sampling details had little impact on our results (Table S3).

In our coalescent model, selective neutrality was assumed among lineages, however much of the effect of selection will be captured by the effective population size parameter [4]. Additionally, effective population sizes and rates of migration were assumed constant over time. However, given the strong seasonality exhibited by influenza [1], we expect variation in the rates of migration and coalescence over the course of a year. By assuming constant rates of coalescence and migration, our estimates mask such rate variation. It is nonetheless noteworthy that a relatively high rate of migration was inferred from the USA to Oceania, despite their strongly asynchronous epidemic dynamics. We might expect that, during the Southern Hemisphere summer when influenza is common in the USA, migration events from the USA into Oceania should be rare, as seasonal forcing should prevent the newly emigrated lineage from getting a foothold in Oceania. Furthermore, we might expect most migration from the USA into Oceania to occur during the Northern Hemisphere spring/Southern Hemisphere fall, when levels of seasonality and immunity are most favorable to emigrant lineages. More complex statistical models will help explore the nuances of the seasonal migration dynamics of the disease.

For each of the 100 bootstrap replicates, fifty MCMC chains were run for 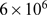 steps each, sampling genealogies and parameter values every 10,000 steps. The first 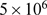 steps of each chain were removed as ‘burn-in.’ Convergence was assessed visually and through comparison among chains using the Gelman-Rubin convergence statistic. We combined the remaining samples from each chain to give a total of 5,000 samples for each of the resampled replicates.

We performed a number of additional checks to confirm that our results were robust to the details of the analysis. Instead of equal sampling from region, we sampled from each region in proportion to its human population size (Table S5). We performed a number of analyses adjusting the migration rate prior. We found more variation in the migration network using larger priors, but the details of connectivity were highly similar (Table S6). Larger priors resulted in a larger USA contribution, suggesting that our choice of smaller prior is a conservative one. We also performed a number of analyses using alternative regional groupings, for example dropping South America and splitting China into two regions: China and Hong Kong (Table S7). In all cases, we still find support for a global meta-population model in which the USA plays a strong role. In addition, the relative rates of migration between regions were similar between analyses.

Nevertheless, despite our best efforts to control for sampling effects, it remains possible that overlooked sampling details may have influenced our results. With progress in worldwide surveillance and sequencing technology, constructing a truly representative sample of influenza should eventually become tractable.

### Genealogical reconstruction

For reconstruction of genealogical trees we cut down the full 4355 sequences to 2165 sequences by taking at most 10 sequences per month from each region. This served to make the analysis more computationally feasible, while retaining as much temporal information as possible. Sample counts from the USA, and to a lesser extent Japan and Oceania, were decremented by this method, while other regions were affected only slightly (Table S1). In our analysis of these sequences, we held migration rates and effective population sizes constant at the levels estimated from the preceding resampling analysis.

The trunk of the influenza genealogy can only be identified in retrospect. All branches in trees sampled by Migrate are labeled with the demes they occupy. To assess deme-specific contributions to the trunk, we first extracted the trunk from the genealogy. This was done by taking random samples present between 2007 and 2009 and tracing their ancestry backwards in time. Thus, each random sample gives a slightly different trunk. Farther in the past, all samples share the same lineage as the trunk, while closer to the present samples may differ in which lineage they consider to be the trunk. Uncertainty is further encapsulated by analyzing the trunks of a sample of genealogies, rather than just using a single tree. Across all sampled trunks, we calculated the mean and credible interval for the proportion of each trunk belonging to a particular geographic region (Table 2). The temporal dynamics were assessed in a similar fashion, calculating the proportion of sampled genealogies belonging to a particular region at a particular point in time (Figure 3). Trunk extraction and processing was performing using the program PACT, which is freely available from the author's website (http://www.trevorbedford.com/pact).

Because of the larger dataset, MCMC chains had to run for significantly longer than in the previous analysis. Four MCMC chains were run for 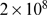 steps each. The first 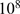 steps of each chain were removed as burn-in. Genealogical trees were sampled every 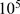 steps. Combining the remaining data left a sample of 4000 genealogical trees in which to perform trunk reconstruction.

### Epidemiological simulations

To validate our methods, we implemented a stochastic, multi-strain, multi-deme susceptible-infected-recovered-susceptible (SIRS) model. Three host populations (North, Tropics and South) were simulated with epidemiological parameters derived from influenza A (H3N2). Here, we refer to these populations as demes. In these simulations, the North and the South were seasonally forced, so that every summer infection dies out. We tested two ecological scenarios. In the source-sink model, infected hosts within the Tropics can contact hosts in the North and in the South. Infected individuals in the North and the South never contact susceptibles outside their own demes. The second model was an equal contact model, where bidirectional migration occurred between all demes. We suggest that what is most important here is the concordance between simulation parameters and our estimates of these parameters, rather than that the simulation model perfectly reflect reality.

The epidemiological model was run for 50 simulated years with the first 40 years removed as ‘burn-in’ to allow genetic diversity and population dynamics to equilibrate. All epidemiological and demographic parameters, except contact rates, were identical between demes. Host population sizes in each deme were kept constant at 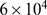 individuals with per-capita birth and death rates of 30 years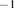. Strains had an intrinsic reproductive rate 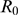 of 2 [26], [27], an average duration of infection of 5 days [28], and an average duration of immunity of 2 years [29]. The North and South populations were seasonally forced using a sinusoidal function with amplitude 0.4, and thus 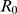 varied from 1.2 to 2.8. A strain was defined as a sequence of 1000 bases. The rate of mutation 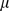 was 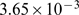 substitutions per site per year. In the source-sink model, the per capita probability of transmission from the Tropics to the North and from the Tropics to the South was 0.005 of the rate of within-deme transmission. In the equal contact model, between-deme transmission was 0.005 of the rate of within-deme transmission for each pair of demes. In each case, this translates to an expectation of 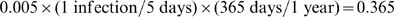 migration events per lineage per year.

Under these parameters, infection in the Tropics reaches an endemic equilibrium, while infection in the temperate regions shows seasonal epidemics (Figure 4). In the Tropics, there was an average of 248.8 and 251.0 infected individuals on any given day for the source-sink model and the equal contact model respectively (Table 3). This is the population size of each regional virus population. The generation time is given by duration of infection and is equal to 5 days, or 73 generations per year.

Five hundred sequences were sampled from each deme over the final 10 years of the simulation (Figure 4). Sampling was proportional to the size of the regional population, so that the seasonality of the North and the South is reflected in the temporal distribution of samples. We ran Migrate v3.0.8 [14], [20] with these samples to assess deme-specific rates of coalescence and migration. Estimates of effective population size of the Tropics agreed well with the true population size in the simulations of both models (Table 3). Estimates of the effective population size in the North and South are lower than the mean census population size owing to the large fluctuations in census size over time (Figure 4). Estimated rates of migration were highly compatible with the contact rates specified in the model (Table 3). In cases where there the true contact rate was 0.365, the estimated contact rates ranged from 0.37 to 0.39. In cases where the true contact rate was 0.0, the estimated contact rates ranged from 0.01 to 0.06.

The overall appearance of the genealogical trees reconstructed from simulation data (Figure 4) was very close to the reconstructed influenza genealogy (Figure 2). In the source-sink model, we see that seasonal epidemics in the North and in the South never contribute viral lineages to the trunk, while in the equal contact model, seasonal epidemics sometimes lead to chains of transmission in the Tropics and sometimes lead directly to the next seasonal epidemic in the other hemisphere (Figure 4). Here, the reconstruction of the genealogy trunk was in agreement with our expectations. In the source-sink model, the location of trunk of the genealogy is estimated to always reside in the Tropics (Figure 4). This makes intuitive sense in that with asymmetric migration only the Tropics may possibly contribute to the long-term evolution of the simulated virus population. In the equal contact model, the location of the trunk varies dynamically over time (Figure 4). Although the Tropics make the largest contribution, both the North and South sometimes comprise the trunk of the genealogy and thus shape long-term viral evolution.

To further confirm that our coalescent inference methodology is robust to sampling details, we performed an additional analysis of simulation data from the source-sink model and from the equal contact model. In this analysis, sampling intensity was intentionally skewed with 500 samples were taken from the North and South, but only 100 samples taken from the Tropics. Results from this analysis were highly similar to the analysis with equal sampling intensity (Table S8).

## Supporting Information

Figure S1 Distribution of 4355 influenza A (H3N2) samples across countries of origin. Circles are colored according to our regional partitioning. Circle areas are proportional to sample count of the full dataset before any resampling took place.
(0.26 MB PDF)

Figure S2 Distribution of 4355 influenza A (H3N2) samples over time across regions. Each bin represents a single month. Sample counts are of the full dataset before any resampling took place.
(0.30 MB PDF)

Table S1 Number of sequences used from each geographic region in different stages of the analysis.
(0.03 MB PDF)

Table S2 Regional genetic diversity π arrayed below the diagonal, measured in terms of 10^−3^ substitutions per site, and regional *F_ST_* arrayed above diagonal, with 95% confidence intervals determined by 1000 bootstrap replicates.
(0.09 MB PDF)

Table S3 Estimates for immigration (columns) and emigration (rows) rates between each pair of regions measured in terms of migration events per lineage per year.
(0.03 MB PDF)

Table S4 Estimates for the timescale of coalescence (in years) and the effective population size (in number of individuals) of each region, assuming overlapping generations and an infectious period (generation time) of 5 days.
(0.04 MB PDF)

Table S5 Estimates using proportional sampling for immigration (columns) and emigration (rows) rates between each pair of regions measured in terms of migration events per lineage per year.
(0.03 MB PDF)

Table S6 Estimates using a 1000-fold larger prior for immigration (columns) and emigration (rows) rates between each pair of regions measured in terms of migration events per lineage per year.
(0.03 MB PDF)

Table S7 Estimates using an alternative geographical grouping for immigration (columns) and emigration (rows) rates between each pair of regions measured in terms of migration events per lineage per year.
(0.03 MB PDF)

Table S8 Mean estimates and 95% credible intervals for effective population size *N_e_*, rate of migration *m* and trunk proportion for each region in simulated data sets based on 500 samples from the North, 500 samples from the South and 100 samples from the Tropics over a 10 year period.
(0.06 MB PDF)

## References

[1] World Health Organization (2009). Fact sheet Number 211.. http://www.who.int/mediacentre/factsheets/fs211/en/.

[2] Nelson MI, Simonsen L, Viboud C, Miller MA, Holmes EC (2007). Phylogenetic analysis reveals the global migration of seasonal influenza A viruses.. PLoS Pathog.

[3] Russell CA, Jones TC, Barr IG, Cox NJ, Garten RJ (2008). The global circulation of seasonal influenza A (H3N2) viruses.. Science.

[4] Rambaut A, Pybus OG, Nelson MI, Viboud C, Taubenberger JK (2008). The genomic and epidemiological dynamics of human influenza A virus.. Nature.

[5] Ferguson NM, Galvani AP, Bush RM (2003). Ecological and immunological determinants of influenza evolution.. Nature.

[6] Cargill M, Altshuler D, Ireland J, Sklar P, Ardlie K (1999). Characterization of single-nucleotide polymorphisms in coding regions of human genes.. Nat Genet.

[7] Brown AJ (1997). Analysis of HIV-1 env gene sequences reveals evidence for a low effective number in the viral population.. Proc Natl Acad Sci U S A.

[8] Nelson MI, Simonsen L, Viboud C, Miller MA, Taylor J (2006). Stochastic processes are key determinants of short-term evolution in influenza A virus.. PLoS Pathog.

[9] Hudson RR, Slatkin M, Maddison WP (1992). Estimation of levels of gene flow from DNA sequence data.. Genetics.

[10] Shriver MD, Mei R, Parra EJ, Sonpar V, Halder I (2005). Large-scale SNP analysis reveals clustered and continuous patterns of human genetic variation.. Hum Genomics.

[11] Kingman JFC (1982). The coalescent.. Stochast Proc Appl.

[12] Notohara M (1990). The coalescent and the genealogical process in geographically structured population.. J Math Biol.

[13] Drummond AJ, Nicholls GK, Rodrigo AG, Solomon W (2002). Estimating mutation parameters, population history and genealogy simultaneously from temporally spaced sequence data.. Genetics.

[14] Beerli P, Felsenstein J (2001). Maximum likelihood estimation of a migration matrix and effective population sizes in *n* subpopulations by using a coalescent approach.. Proc Natl Acad Sci U S A.

[15] Hufnagel L, Brockmann D, Geisel T (2004). Forecast and control of epidemics in a globalized world.. Proc Natl Acad Sci U S A.

[16] Fitch WM, Bush RM, Bender CA, Cox NJ (1997). Long term trends in the evolution of H(3) HA1 human influenza type A.. Proc Natl Acad Sci U S A.

[17] Nelson MI, Holmes EC (2007). The evolution of epidemic influenza.. Nat Rev Genet.

[18] Koelle K, Cobey S, Grenfell B, Pascual M (2006). Epochal evolution shapes the phylodynamics of interpandemic influenza A (H3N2) in humans.. Science.

[19] Wolf YI, Viboud C, Holmes EC, Koonin EV, Lipman DJ (2006). Long intervals of stasis punctuated by bursts of positive selection in the seasonal evolution of influenza A virus.. Biol Direct.

[20] Beerli P (2006). Comparison of bayesian and maximum-likelihood inference of population genetic parameters.. Bioinformatics.

[21] Maruyama T, Kimura M (1980). Genetic variability and effective population size when local extinction and recolonization of subpopulations are frequent.. Proc Natl Acad Sci U S A.

[22] Bao Y, Bolotov P, Dernovoy D, Kiryutin B, Zaslavsky L (2008). The influenza virus resource at the national center for biotechnology information.. J Virol.

[23] Edgar RC (2004). MUSCLE: multiple sequence alignment with high accuracy and high throughput.. Nucleic Acids Res.

[24] Drummond AJ, Rambaut A (2007). BEAST: Bayesian evolutionary analysis by sampling trees.. BMC Evol Biol.

[25] Hasegawa M, Kishino H, Yano T (1985). Dating of the human-ape splitting by a molecular clock of mitochondrial dna.. J Mol Evol.

[26] Gani R, Hughes H, Fleming D, Griffin T, Medlock J (2005). Potential impact of antiviral drug use during influenza pandemic.. Emerg Infect Dis.

[27] Cauchemez S, Valleron AJ, Boelle PY, Flahault A, Ferguson NM (2008). Estimating the impact of school closure on influenza transmission from Sentinel data.. Nature.

[28] Carrat F, Vergu E, Ferguson NM, Lemaitre M, Cauchemez S (2008). Time lines of infection and disease in human influenza: a review of volunteer challenge studies.. Am J Epidemiol.

[29] Smith DJ, Lapedes AS, de Jong JC, Bestebroer TM, Rimmelzwaan GF (2004). Mapping the antigenic and genetic evolution of influenza virus.. Science.

[30] Borgatti S (2005). Centrality and network flow.. Social Networks.

