# Supplementary material for: Global Migration Dynamics Underlie Evolution and Persistence of Human Influenza A (H3N2)
Source: PLoS Pathog. 2010 May 27;6(5):e1000918. doi: 10.1371/journal.ppat.1000918 (PMC2877742; doi:10.1371/journal.ppat.1000918)
Supplement: Figure S1 — Distribution of 4355 influenza A (H3N2) samples across countries of origin. Circles are colored according to our regional partitioning. Circle areas are proportional to sample count of the full dataset before any resampling took place. (0.26 MB PDF) [file ppat.1000918.s001.pdf]

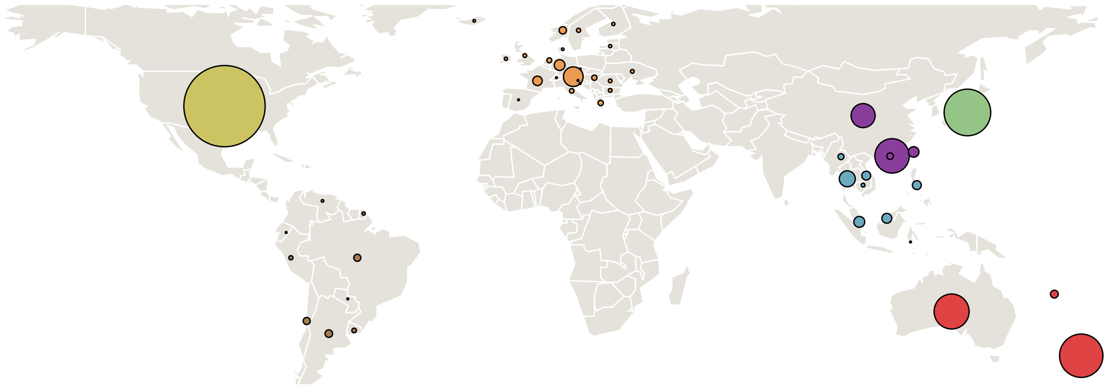

Figure S1. Distribution of 4355 influenza A (H3N2) samples across countries of origin. Circles are colored according to our regional partitioning. Circle areas are proportional to sample count of the full dataset before any resampling took place.
