# Supplementary material for: Global Migration Dynamics Underlie Evolution and Persistence of Human Influenza A (H3N2)
Source: PLoS Pathog. 2010 May 27;6(5):e1000918. doi: 10.1371/journal.ppat.1000918 (PMC2877742; doi:10.1371/journal.ppat.1000918)
Supplement: Figure S2 — Distribution of 4355 influenza A (H3N2) samples over time across regions. Each bin represents a single month. Sample counts are of the full dataset before any resampling took place. (0.30 MB PDF) [file ppat.1000918.s002.pdf]

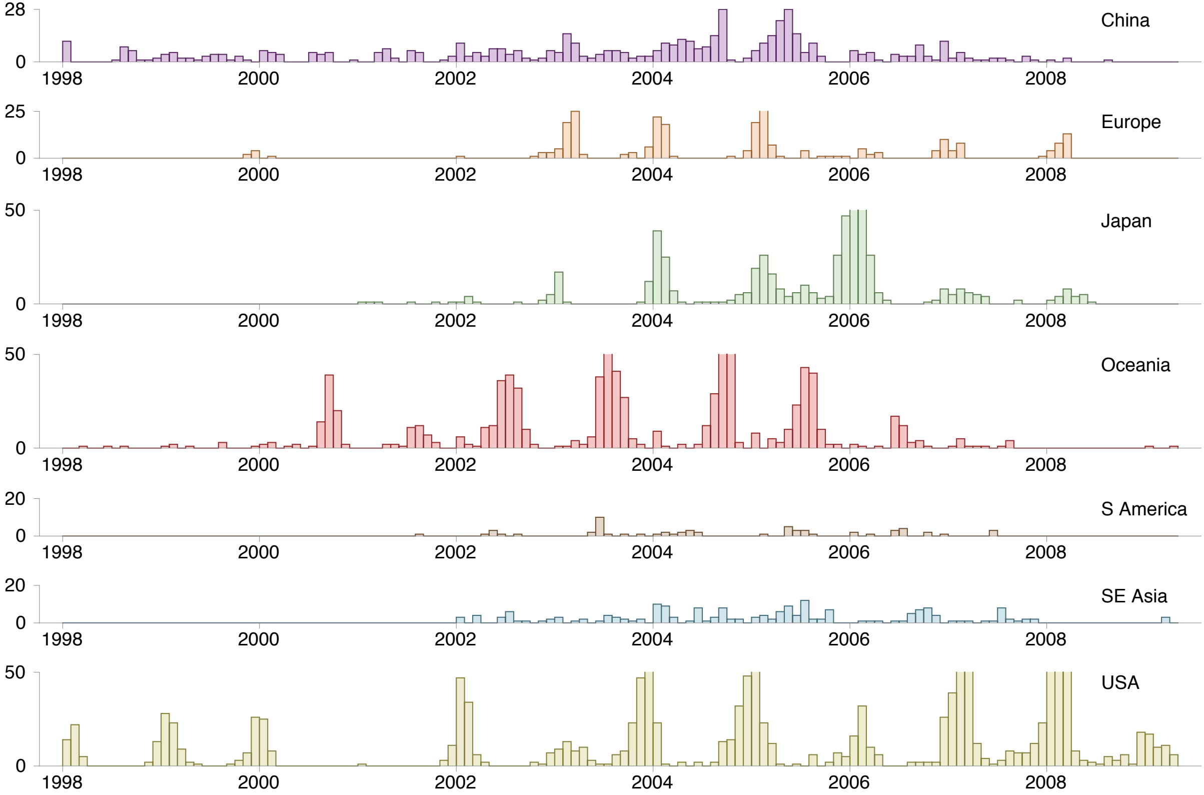

Figure S2. Distribution of 4355 influenza A (H3N2) samples over time across regions. Each bin represents a single month. Sample counts are of the full dataset before any resampling took place.
