# Supplementary material for: Global Migration Dynamics Underlie Evolution and Persistence of Human Influenza A (H3N2)
Source: PLoS Pathog. 2010 May 27;6(5):e1000918. doi: 10.1371/journal.ppat.1000918 (PMC2877742; doi:10.1371/journal.ppat.1000918)
Supplement: Table S1 — Number of sequences used from each geographic region in different stages of the analysis. (0.03 MB PDF) [file ppat.1000918.s003.pdf]

Table S1. Number of sequences used from each geographic region in different stages of the analysis.

	Diversity estimates	Equal sampling	Proportional sampling	Genealogical estimates
China	534	61	206	463
Europe	248	61	94	174
Japan	603	61	20	280
Oceania	881	61	6	394
South America	62	61	61	62
Southeast Asia	190	61	90	188
USA	1837	61	49	604
Total	4355	427	526	2165
