# Supplementary material for: Global Migration Dynamics Underlie Evolution and Persistence of Human Influenza A (H3N2)
Source: PLoS Pathog. 2010 May 27;6(5):e1000918. doi: 10.1371/journal.ppat.1000918 (PMC2877742; doi:10.1371/journal.ppat.1000918)
Supplement: Table S2 — Regional genetic diversity π arrayed below the diagonal, measured in terms of 10−3 substitutions per site, and regional FST arrayed above diagonal, with 95% confidence intervals determined by 1000 bootstrap replicates. (0.09 MB PDF) [file ppat.1000918.s004.pdf]

Table S2. Regional genetic diversity π arrayed below the diagonal, measured in terms of 10^{-3} substitutions per site, and regional F_{ST} arrayed above diagonal, with 95% confidence intervals determined by 1000 bootstrap replicates.

	China	Europe	Japan	Oceania	S America	SE Asia	USA
China	6.1 (5.8, 6.5)	0.50 (0.46, 0.54)	0.08 (0.04, 0.12)	0.50 (0.47, 0.52)	0.26 (0.05, 0.43)	0.24 (0.16, 0.30)	0.46 (0.43, 0.48)
Europe	16.5 (15.6, 17.6)	10.3 (9.4, 11.2)	0.07 (0.02, 0.12)	0.34 (0.26, 0.41)	−0.28 (−0.73, 0.05)	0.17 (0.07, 0.25)	−0.02 (−0.10, 0.05)
Japan	8.9 (8.5, 9.2)	11.0 (10.6, 11.4)	10.1 (9.9, 10.3)	0.22 (0.18, 0.26)	−0.20 (−0.48, 0.03)	−0.05 (−0.15, 0.04)	0.16 (0.14, 0.17)
Oceania	11.8 (11.5, 12.1)	12.1 (11.1, 13.2)	10.2 (9.8, 10.6)	5.7 (5.5, 5.9)	0.37 (0.21, 0.51)	0.38 (0.33, 0.43)	0.32 (0.29, 0.35)
S America	15.0 (13.6, 16.2)	9.9 (8.1, 12.1)	10.9 (10.1, 11.7)	17.4 (16.1, 18.7)	16.0 (11.1, 21.2)	0.13 (−0.10, 0.33)	−0.06 (−0.43, 0.23)
SE Asia	9.6 (9.1, 10.0)	11.3 (10.5, 12.2)	8.5 (8.0, 9.0)	11.4 (10.9, 11.9)	14.1 (12.5, 15.8)	8.5 (7.6, 9.4)	0.36 (0.31, 0.41)
USA	10.5 (10.1, 10.8)	7.6 (7.3, 7.9)	9.1 (9.0, 9.3)	8.1 (7.8, 8.4)	10.1 (8.3, 12.1)	10.8 (10.3, 11.3)	5.3 (5.2, 5.3)
