# Supplementary material for: Global Migration Dynamics Underlie Evolution and Persistence of Human Influenza A (H3N2)
Source: PLoS Pathog. 2010 May 27;6(5):e1000918. doi: 10.1371/journal.ppat.1000918 (PMC2877742; doi:10.1371/journal.ppat.1000918)
Supplement: Table S3 — Estimates for immigration (columns) and emigration (rows) rates between each pair of regions measured in terms of migration events per lineage per year. (0.03 MB PDF) [file ppat.1000918.s005.pdf]

Table S3. Estimates for immigration (columns) and emigration (rows) rates between each pair of regions measured in terms of migration events per lineage per year.

	China	Europe	Japan	Oceania	S America	SE Asia	USA
China	–	.07 (.03, .15)	.29 (.14, .42)	.15 (.07, .30)	.06 (.03, .10)	.30 (.11, .51)	.19 (.06, .34)
Europe	.06 (.03, .13)	–	.06 (.02, .12)	.16 (.07, .34)	.08 (.04, .13)	.11 (.05, .23)	.12 (.04, .29)
Japan	.10 (.04, .30)	.07 (.03, .15)	–	.13 (.07, .23)	.04 (.02, .08)	.10 (.04, .21)	.06 (.03, .15)
Oceania	.06 (.03, .13)	.13 (.06, .22)	.07 (.03, .14)	–	.03 (.02, .06)	.10 (.04, .25)	.16 (.05, .41)
S America	.04 (.02, .08)	.05 (.03, .10)	.04 (.02, .07)	.08 (.04, .14)	–	.04 (.02, .06)	.05 (.02, .10)
SE Asia	.27 (.11, .49)	.14 (.06, .25)	.13 (.05, .28)	.23 (.11, .42)	.03 (.02, .07)	–	.11 (.05, .26)
USA	.25 (.10, .47)	.25 (.13, .35)	.19 (.06, .34)	.51 (.20, .89)	.22 (.17, .27)	.20 (.08, .41)	–

Estimates represent means and 95% confidence intervals across 100 resampled replicates.

Sampling was constrained to 61 sequences per deme taken between the years 2002 and 2008.

Migration rates were given an exponential prior with a mean of 0.1 substitutions per site.
