# Supplementary material for: Global Migration Dynamics Underlie Evolution and Persistence of Human Influenza A (H3N2)
Source: PLoS Pathog. 2010 May 27;6(5):e1000918. doi: 10.1371/journal.ppat.1000918 (PMC2877742; doi:10.1371/journal.ppat.1000918)
Supplement: Table S4 — Estimates for the timescale of coalescence (in years) and the effective population size (in number of individuals) of each region, assuming overlapping generations and an infectious period (generation time) of 5 days. (0.04 MB PDF) [file ppat.1000918.s006.pdf]

Table S4. Estimates for the timescale of coalescence (in years) and the effective population size (in number of individuals) of each region, assuming overlapping generations and an infectious period (generation time) of 5 days.

	Timescale of coalescence	Effective population size
China	0.72 (0.50, 1.01)	105.7 (72.8, 147.4)
Europe	1.62 (1.06, 2.23)	236.2 (154.9, 325.1)
Japan	1.69 (0.93, 2.67)	246.4 (136.4, 389.9)
Oceania	0.50 (0.29, 0.78)	72.6 (42.9, 113.4)
South America	2.68 (2.43, 2.97)	391.3 (354.5, 433.3)
Southeast Asia	1.16 (0.74, 1.72)	168.9 (107.5, 251.1)
USA	0.88 (0.62, 1.15)	128.5 (90.8, 167.4)

Estimates represent means and 95% confidence intervals across 100 resampled replicates.

Sampling was constrained to 61 sequences per deme taken between the years 2002 and 2008.

Migration rates were given an exponential prior with a mean of 0.1 substitutions per site.
