# Supplementary material for: Global Migration Dynamics Underlie Evolution and Persistence of Human Influenza A (H3N2)
Source: PLoS Pathog. 2010 May 27;6(5):e1000918. doi: 10.1371/journal.ppat.1000918 (PMC2877742; doi:10.1371/journal.ppat.1000918)
Supplement: Table S5 — Estimates using proportional sampling for immigration (columns) and emigration (rows) rates between each pair of regions measured in terms of migration events per lineage per year. (0.03 MB PDF) [file ppat.1000918.s007.pdf]

Table S5. Estimates using proportional sampling for immigration (columns) and emigration (rows) rates between each pair of regions measured in terms of migration events per lineage per year.

	China	Europe	Japan	Oceania	S America	SE Asia	USA
China	–	0.11	0.43	0.13	0.09	0.38	0.25
Europe	0.06	–	0.05	0.18	0.11	0.17	0.31
Japan	0.10	0.04	–	0.12	0.02	0.05	0.05
Oceania	0.04	0.08	0.05	–	0.05	0.07	0.09
S America	0.03	0.06	0.03	0.07	–	0.04	0.09
SE Asia	0.16	0.15	0.15	0.18	0.04	–	0.13
USA	0.14	0.15	0.08	0.24	0.20	0.13	–

Estimates represent means across 100 resampled replicates.

Sampling was constrained to be proportional to the human population size of each deme, giving 206 samples for China, 94 samples for Europe, 20 samples for Japan, 6 samples for Oceania, 61 samples for Oceania, 90 samples for Southeast Asia and 49 samples for the USA. All samples were taken between the years 2002 and 2008.

Migration rates were given an exponential prior with a mean of 0.1 substitutions per site.
