# Supplementary material for: Global Migration Dynamics Underlie Evolution and Persistence of Human Influenza A (H3N2)
Source: PLoS Pathog. 2010 May 27;6(5):e1000918. doi: 10.1371/journal.ppat.1000918 (PMC2877742; doi:10.1371/journal.ppat.1000918)
Supplement: Table S6 — Estimates using a 1000-fold larger prior for immigration (columns) and emigration (rows) rates between each pair of regions measured in terms of migration events per lineage per year. (0.03 MB PDF) [file ppat.1000918.s008.pdf]

Table S6. Estimates using a 1000-fold larger prior for immigration (columns) and emigration (rows) rates between each pair of regions measured in terms of migration events per lineage per year.

	China	Europe	Japan	Oceania	S America	SE Asia	USA
China	–	0.32	1.83	0.39	0.24	1.93	0.45
Europe	0.10	–	0.31	0.29	0.38	0.32	0.21
Japan	0.23	0.46	–	0.28	0.28	0.68	0.17
Oceania	0.14	0.69	0.40	–	0.28	0.39	0.34
S America	0.08	0.47	0.29	0.19	–	0.27	0.18
SE Asia	0.35	0.45	0.91	0.38	0.28	–	0.21
USA	0.22	0.67	0.46	0.77	1.40	0.55	–

Estimates represent means across 100 resampled replicates.

Sampling was constrained to 61 sequences per deme taken between the years 2002 and 2008.

Migration rates were given an exponential prior with a mean of 100 substitutions per site.
