# Supplementary material for: Global Migration Dynamics Underlie Evolution and Persistence of Human Influenza A (H3N2)
Source: PLoS Pathog. 2010 May 27;6(5):e1000918. doi: 10.1371/journal.ppat.1000918 (PMC2877742; doi:10.1371/journal.ppat.1000918)
Supplement: Table S7 — Estimates using an alternative geographical grouping for immigration (columns) and emigration (rows) rates between each pair of regions measured in terms of migration events per lineage per year. (0.03 MB PDF) [file ppat.1000918.s009.pdf]

Table S7. Estimates using an alternative geographical grouping for immigration (columns) and emigration (rows) rates between each pair of regions measured in terms of migration events per lineage per year.

	China	Europe	Hong Kong	Japan	Oceania	SE Asia	USA
China	–	0.03	0.57	0.34	0.06	0.10	0.13
Europe	0.03	–	0.09	0.11	0.20	0.17	0.15
Hong Kong	0.12	0.04	–	0.17	0.07	0.23	0.07
Japan	0.07	0.08	0.16	–	0.14	0.12	0.08
Oceania	0.04	0.15	0.04	0.10	–	0.13	0.15
SE Asia	0.11	0.11	0.31	0.16	0.20	–	0.06
USA	0.11	0.18	0.07	0.24	0.30	0.17	–

Estimates represent means across 100 resampled replicates.

Sampling was constrained to 175 sequences per deme taken between the years 2002 and 2008.

Migration rates were given an exponential prior with a mean of 0.1 substitutions per site.
