# Supplementary material for: Global Migration Dynamics Underlie Evolution and Persistence of Human Influenza A (H3N2)
Source: PLoS Pathog. 2010 May 27;6(5):e1000918. doi: 10.1371/journal.ppat.1000918 (PMC2877742; doi:10.1371/journal.ppat.1000918)
Supplement: Table S8 — Mean estimates and 95% credible intervals for effective population size Ne, rate of migration m and trunk proportion for each region in simulated data sets based on 500 samples from the North, 500 samples from the South and 100 samples from the Tropics over a 10 year period. (0.06 MB PDF) [file ppat.1000918.s010.pdf]

**Table S8.** Mean estimates and 95% credible intervals for effective population size $N_e$, rate of migration $m$ and trunk proportion for each region in simulated data sets based on 500 samples from the North, 500 samples from the South and 100 samples from the Tropics over a 10 year period.

| | | Source-sink model | Equal contact model |
|---|---|---|---|
| Population size | $N$ | 116.1 (92.4, 152.2) | 126.3 (99.6, 164.6) |
| | $T$ | 165.9 (126.8, 223.2) | 222.8 (188.2, 278.8) |
| | $S$ | 169.6 (139.6, 210.2) | 158.2 (130.8, 201.6) |
| Migration rate | $N \rightarrow T$ | 0.14 (0.01, 0.41) | 0.28 (0.10, 0.48) |
| | $N \rightarrow S$ | 0.02 (0.00, 0.08) | 0.57 (0.31, 0.93) |
| | $T \rightarrow N$ | 0.44 (0.23, 0.72) | 0.35 (0.19, 0.55) |
| | $T \rightarrow S$ | 0.43 (0.23, 0.70) | 0.33 (0.17, 0.53) |
| | $S \rightarrow N$ | 0.03 (0.00, 0.09) | 0.25 (0.06, 0.56) |
| | $S \rightarrow T$ | 0.10 (0.00, 0.29) | 0.34 (0.19, 0.53) |
| Trunk proportion | $N$ | 0.01 (0.00, 0.13) | 0.27 (0.16, 0.39) |
| | $T$ | 0.98 (0.84, 1.00) | 0.55 (0.42, 0.67) |
| | $S$ | 0.01 (0.00, 0.08) | 0.18 (0.06, 0.31) |
